# Carbon Nanopowder-Based Stochastic Sensor for Ultrasensitive Assay of CA 15-3, CEA and HER2 in Whole Blood

**DOI:** 10.3390/nano12183111

**Published:** 2022-09-08

**Authors:** Raluca-Ioana Stefan-van Staden, Oana-Raluca Musat, Damaris-Cristina Gheorghe, Ruxandra-Maria Ilie-Mihai, Jacobus (Koos) Frederick van Staden

**Affiliations:** 1 Laboratory of Electrochemistry and PATLAB, 202 Splaiul Independentei Str., 060021 Bucharest, Romania; 2 Faculty of Chemical Engineering and Biotechnologies, University Politehnica of Bucharest, 011061 Bucharest, Romania

**Keywords:** CA15-3, CEA, HER2, nanocarbon, stochastic microsensor

## Abstract

Two microsensors obtained by the physical immobilization of 5,10,15,20-tetraphenyl-21H,23H-porphine (TPP) and 5,10,15,20-tetrakis (pentafluorophenyl chloride)-21H,23H-iron (III) porphyrin (Fe(TPFPP)Cl) in carbon nanopowder decorated with gold nanoparticles (AuNp) were designed, characterized, validated and used for the molecular recognition and simultaneous ultrasensitive determination of CEA, CA15-3 and HER2 in whole blood. High sensitivities were recorded for both microsensors. Low limits of quantification were recorded for all biomarkers: CEA (12.8 pg mL^−1^ by using Fe(TPFPP)Cl/AuNp, and 190 fg mL^−1^ by using TPP/AuNp), CA 15-3 (100 fU mL^−1^ for both microsensors) and HER2 (3.9 fg mL^−1^ by using Fe(TPFPP)Cl/AuNp, and 35 fg mL^−1^ by using TPP/AuNp). A very good correlation between the results obtained using the proposed microsensors and ELISA, certified by the Student t-test, proves that the screening test can be used for ultrasensitive assays of the three biomarkers in whole blood.

## 1. Introduction

CA 15-3, CEA and HER-2 play the role of prognostic biomarkers and can facilitate personalized treatment for breast cancer. Early diagnosed breast cancer, as well as prescribing a personalized treatment for the confirmed breast cancer patient, have a major impact on the patient’s health, as well as being closely related to test results and rapid, reliable and accurate screening [1,2]. CA15-3 is useful for the early detection of tumor recurrence in patients previously treated for stage II and III breast cancer, with no clinical signs of disease activity. The combined determination of CA 15-3 and CEA may increase the sensitivity of recurrent tumor detection [3]. CA 15-3 and CEA levels in breast cancer proved to be sensitive biomarkers for the evaluation of patients with breast cancer [4]. HER2-positive breast cancers tend to be more aggressive than HER2-negative breast cancers. Along with tumor grade and cancer stage, HER2 status helps to determine the treatment options [5].

ELISA (based on assay kits available from many companies) remains the standard method of choice for the assay of CA15-3, CEA and HER2, although, for HER2, the immunohistochemistry method is preferred, especially when determined from tumor tissue. Numerous methods were proposed for the assay of CA15-3, CEA, and HER2 (Table S1) [6,7,8,9,10,11,12,13,14,15]. The importance of the utilization of nanomaterials in the biomedical analysis of CA15-3 was shown in a review article [15]. Biosensors and immunosensors were also proposed for the assay of these biomarkers (Table S1), but none of the papers reported the simultaneous determination of CA15-3, CEA and HER2.

Therefore, this paper proposes two new stochastic microsensors based on carbon nanopowder (nC) modified with gold nanoparticles (AuNp) and two porphyrins—5,10,15,20-tetraphenyl-21H,23H-porphine (TPP) and 5,10,15,20-tetrakis (pentafluorophenyl chlo-ride)-21H,23H-iron (III) porphyrin (Fe(TPFPP)Cl)—for the simultaneous assay of CEA, CA15-3 and HER2, because a screening test based on their simultaneous assay will provide more information about the diagnostic and personalized treatment of breast cancer. The novelty of the work, besides the new design used for the stochastic microsensors proposed for the assay of CEA, CA 15-3 and HER2, is the utilization of only one tool for the simultaneous assay of the three biomarkers.

The utilization of gold nanoparticles will increase the conductivity of the carbon nanopowder (the matrix used for the active side of the stochastic sensor), while the porphyrin materials will provide the necessary channels (formed within the molecular aggregates) for stochastic sensing [16]. The selection of the stochastic sensor type for these molecules’ assay in whole blood was made because the stochastic sensors are able to perform a reliably qualitative and quantitative assay of biomarkers in whole blood [17,18,19,20,21]. The mechanism of current development for stochastic sensors is based on channel conductivity. The molecule enters the channel and the current drops to zero until the whole molecule is inside the channel—the time needed to enter the channel depends on the size, volume, conformation, unfolding capacity (if it is proteine) and velocity driven by the applied potential, and it is known as the signature of the molecule, denoted as the t_off_ value on the diagrams. Meanwhile, in the channel, redox processes take place, in an equilibrium time denoted as t_on_ on the diagrams; the t_on_ value is read between two t_off_ values, and it depends on the concentration of the molecule in the biological fluid in which the molecule is determined.

## 2. Materials and Methods

All chemicals were of analytical grade. Gold nanoparticle suspension, CA15-3, CEA and HER2 were purchased from Sigma Aldrich (St. Louis, MO, USA), and the paraffin oil was purchased from Fluka (Buchs, Switzerland). Deionized water was used for the preparation of the solutions used in the experiments. All CA15-3, CEA and HER2 solutions were prepared in phosphate buffer solution (PBS, pH = 7.40). When not in use, the solutions were kept at a temperature of –20 °C. 

All electrochemical measurements were performed connecting a computer with the GPES software 4.9 to an AUTOLAB/PGSTAT 302 N (Metrohm, Utrecht, The Netherlands). The electrochemical cell comprised three electrodes: the reference electrode (Ag/AgCl), the counter electrode (Pt) and the working electrode (the stochastic microsensor). All measurements were carried out at 25 °C. A chronoamperometric method was used for the measurements of t_on_ and t_off_ values, at a constant potential (125 mV vs. Ag/AgCl).

For microsensors’ design, 100 mg of carbon nanopowder was mixed with 10 µL gold nanoparticle suspension and paraffin oil was added until a paste was obtained. The paste was divided into two equal parts, and to each was added 50μL of one of the following porphyrins: 5,10,15,20-tetraphenyl-21H,23H-porphine (TPP/AuNp) or 5,10,15,20-tetrakis (pentafluorophenyl chloride)-21H,23H-iron (III) porphyrin (Fe(TPFPP)Cl/AuNp). Silver wire served as contact between the paste and the external circuit. Each modified paste was placed in a non-conducting (3D-printed in the laboratory) plastic tube with an internal diameter of 10μm and a length of 5 mm. The stochastic microsensors were washed with deionized water and dried between measurements. When not in use, they were kept in a dry place.

**Figure 1:**
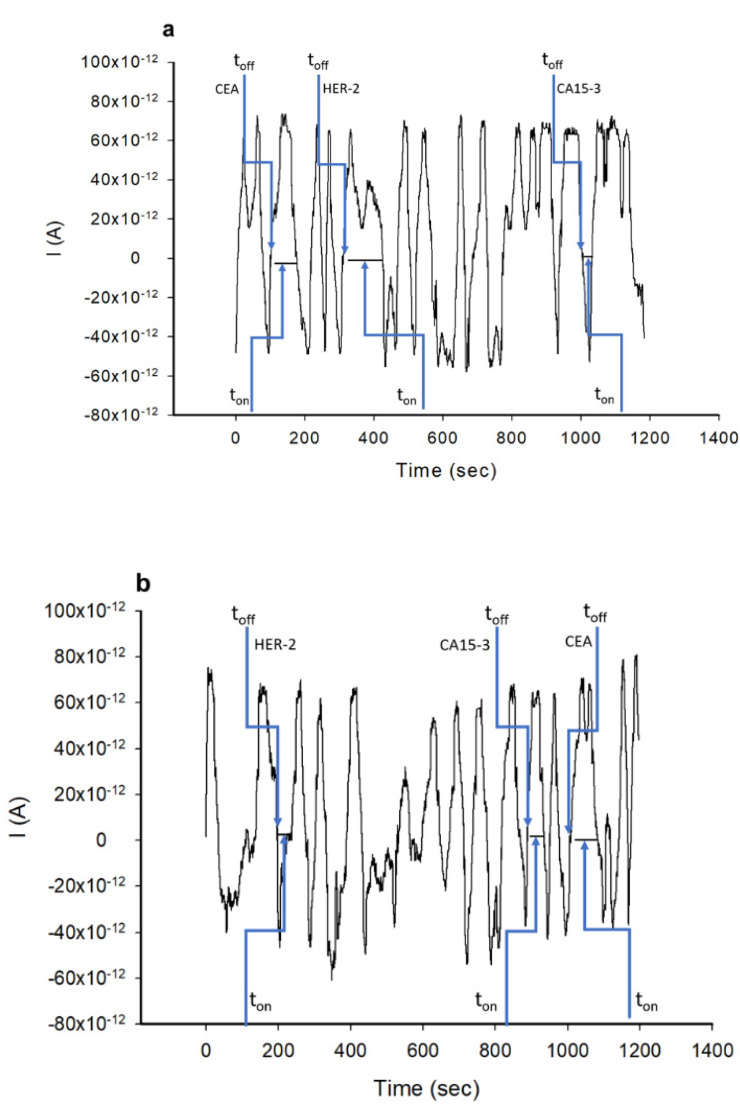
Pattern recognition of CA15-3, CEA and HER2 in whole blood samples, using stochastic microsensors based on (**a**) Fe(TPFPP)Cl/AuNp and (**b**) TPP/AuNp.

The stochastic method was carried out at 25 °C. A chronoamperometric method was used for the measurements of t_on_ and t_off_ at a constant potential (125 mV vs. Ag/AgCl). The value of the applied potential (125 mV vs. Ag/AgCl) was determined experimentally; potentials between 0.05 and 250 mV were applied; the best shape of the stochastic signal was obtained when a potential of 125 mV bs Ag/AgCl was applied. Based on the value of t_off_, the analyte was identified in the diagrams recorded with the stochastic microsensors, and then the value of t_on_ was read and used for the determination of the concentration of each biomarker (Figure 1). The unknown concentrations of CA 15-3, CEA and HER2 in whole blood samples were determined from the calibration equations (1/t_on_ = a + b × C_biomarker_) and recorded with each of the sensors for each of the biomarkers.

The whole blood samples were obtained from the University Hospital Bucharest (approval of the ethics committee No. 11/2013). These samples were obtained from confirmed patients with breast cancer. The biological samples did not require any pretreatment before the measurements. The electrochemical cell was loaded with biological sample, and after recording the diagram and identifying the signatures of CA15-3, CEA and HER2, the unknown concentrations of the biomarkers in the whole blood samples were determined utilizing the stochastic method described above.

## 3. Results and Discussion

### 3.1. Response Characteristics of the Stochastic Microsensors

The response characteristics of the proposed microsensors are shown in Table 1. All response characteristics were determined at 25 °C, when a potential of 125 mV versus Ag/AgCl was applied. First of all, different signatures (t_off_ values) were recorded for CA15-3, CEA and HER2 for each of the stochastic microsenors, proving that the three biomarkers can be determined simultaneously in whole blood samples.

All linear concentration ranges are wide, making possible the determination of these biomarkers at any of the stages of breast cancer. The sensitivities are also very high; higher sensitivities were recorded for the assay of CEA and HER2 when the sensor based on TPP/AuNp was used. Compared with the latest sensors used for the assay of CA15-3, CEA and HER2 (Table S1), one can conclude that the TPP/AuNp-based sensor exhibited the lowest limit of determination (fg mL^−1^ magnitude order), and for the assay of HER2, the Fe(TPFPP)Cl/AuNp-based sensor exhibited the lower limit of determination. For the assay of CA15-3, although the limits of determination are higher than those reported earlier [9,10,11], the proposed sensors can be used for the assay of CA15-3 without any processing of the sample, with the linear concentration range covering patients with breast cancer at any stage of illness. The advantages of the proposed sensors versus those shown in Table S1 are also the following: they can perform the simultaneous detection of the three biomarkers; no sampling is needed before the measurements; reliable qualitative analysis of each biomarker is immediately followed by its quantitative analysis.

### 3.2. Stability and Reproducibility Measurements

Ten stochastic sensors for each of the two types (TPP/AuNp and Fe(TPFPP)Cl/AuNp) were designed, and measurements were performed daily for one month. The measurement for each type of sensor proved that there were no significative changes in the sensitivity, its variation being, for each type, lower than 0.12%; this proved the reproducibility of the design of each type of stochastic sensor. After 30 days of measurements, the variation in the sensitivities recorded for the TPP/AuNp-based stochastic sensor was less than 0.11%, while, for the Fe(TPFPP)Cl/AuNp-based stochastic sensor, it was less than 0.08%; this proved that the sensors are stable for at least one month, when daily measurements are performed.

### 3.3. Selectivity of the Stochastic Microsensors

The selectivity of the stochastic microsensors is given by the difference between the signatures (t_off_ values) recorded for CA15-3, CEA and HER2 and those obtained for other biomarkers/substances from the biological samples. The possible interfering species selected were p53, Ki67, maspin, CA19-9, ascorbic acid, dopamine and uric acid.

Results, shown in Table 2, proved that none of the supposed interfering species interfered with the simultaneous assay of CA15-3, CEA and HER2.

### 3.4. Ultrasensitive Simutaneous Determination of CA15-3, CEA and HER2 in Whole Blood

**Figure 2:**
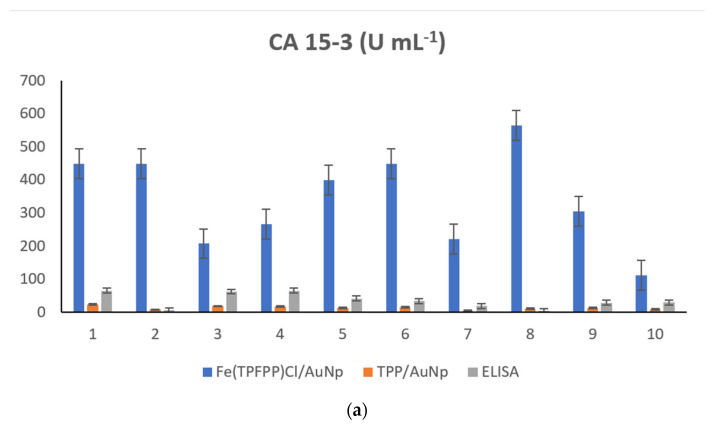

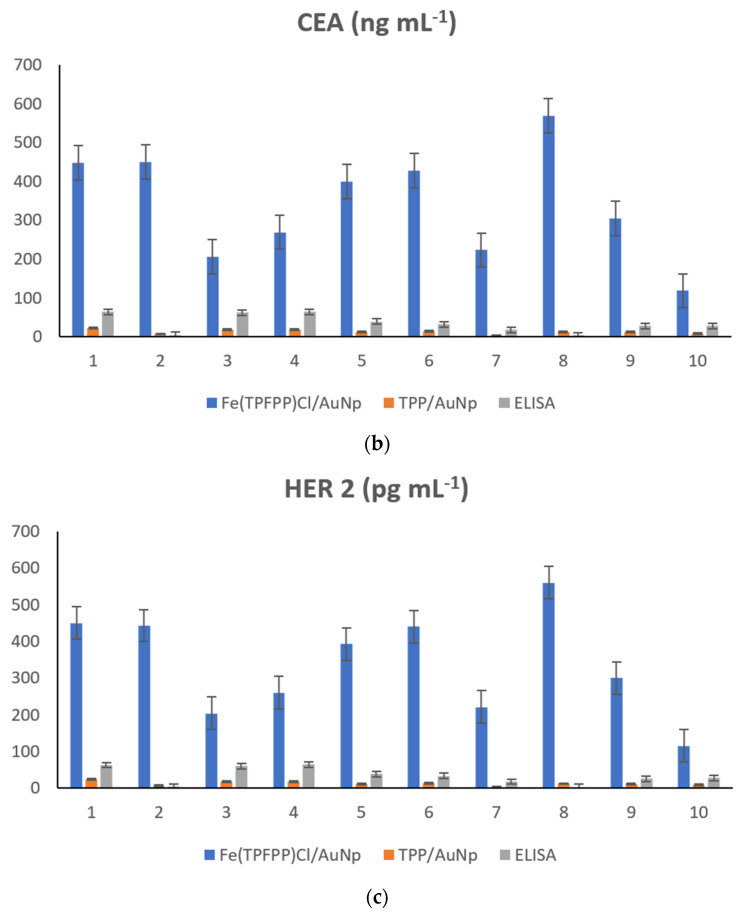
Quantitative determination of (**a**) CA15-3, (**b**) CEA and (**c**) HER2 in whole blood samples, using stochastic microsensors based on Fe(TPFPP)Cl/AuNp and TPP/AuNp, and the standard method—ELISA.

Ten whole blood samples from patients confirmed with breast cancer were screened using the two stochastic microsensors. Shortly after reading the t_off_ values, in between two t_off_ values, the corresponding t_on_ values were read. The t_on_ values were used to determine the concentrations of CA15-3, CEA and HER2 in the whole blood samples, according to the stochastic method described above. The results obtained after the screening of whole blood samples are shown in Table S2 and Figure 2.

Very good correlations between the results obtained using the two stochastic microsensors were obtained. A paired t-test was also performed at a 99.00% confidence level (tabulated theoretical t-value: 4.032) for each biomarker. All calculated t-values were less than the tabulated value, proving that there was no statistically significant difference between the results obtained using the two stochastic microsensors and ELISA (the standard method used for the assay of these biomarkers in whole blood samples) (Table S2, Figure 2). Accordingly, the proposed stochastic microsensors can be reliably used for the ultrasensitive, simultaneous determination of CA15-3, CEA and HER2 in whole blood samples.

## 4. Conclusions

The proposed stochastic microsensors were used for the simultaneous assay of CA15-3, CEA and HER2 in whole blood samples. Their linear working concentration ranges cover patients with breast cancer at any stage of the illness, with determinations being performed with high sensitivity. The screening test based on the utilization of the two microsensors as screening tools may be used for the early detection of breast cancer, for determining the need for personalized treatment, as well as for the determination of the efficiency of the breast cancer treatment.

## Figures and Tables

**Table 1:** Response characteristics of the stochastic sensors used for the assay of CA15-3, CEA and HER 2.

Stochastic Microsensor Based on nC	Signature t_off_ (s)	Linear Concentration Range	Calibration Equations; Correlation Coefficient, r *	Sensitivity	LOQ
Fe(TPFPP)Cl/AuNp	CA15-3 *				
	4.7	1.00 × 10^−7^ − 1.00 × 10^3^	1/t_on_ = 0.03 + 5.80 × 10^3^ × C r = 0.9998	5.80 × 10^3^	1.00 × 10^−7^
	CEA **				
	0.6	1.28 × 10^−5^ − 2.00 × 10^−1^	1/t_on_ = 0.04 + 6.16 × 10 × C r = 0.9993	6.16 × 10	1.28 × 10^−5^
			HER 2 **		
	2.6	3.90 × 10^−9^ − 3.90 × 10^−5^	1/t_on_ = 0.02 + 1.43 × 10^5^ × C r = 0.9999	1.43 × 10^5^	3.90 × 10^−9^
TPP/AuNp	CA15-3 *				
	6.8	1.00 × 10^−7^ − 1.00 × 10^3^	1/t_on_ = 0.04 + 2.32 × 10^3^ × C r = 0.9994	2.32 × 10^3^	1.00 × 10^−7^
	CEA **				
	1.9	1.00 × 10^−7^ − 1.00	1/t_on_ = 0.03 + 1.90 × 10^4^ × C r = 0.9997	1.90 × 10^4^	1.00 × 10^−7^
			HER 2 **		
	1.3	3.50 × 10^−8^ − 3.90 × 10^−5^	1/t_on_ = 0.03 + 3.53 × 10^4^ × C r = 0.9986	3.53 × 10^4^	3.50 × 10^−8^

* <C> = U mL^−1^; <t_on_> = s; <Sensitivity> = s^−1^ U^−1^ mL; ** <C> = µg mL^−1^; <t_on_> = s; <Sensitivity> = s^−1^ μg^−1^ mL; LOQ—limit of quantification.

**Table 2:** Selectivity of the stochastic microsensors.

	Signature (s)									
Stochastic Microsensor Based on nC	CA15-3	CEA	HER2	Maspin	Ki67	CA19-9	p53	Ascorbic Acid	Dopamine	Uric Acid
Fe(TPFPP)Cl/AuNp	4.7	0.6	2.6	2.0	1.3	3.0	3.5	0.2	1.7	3.2
TPP/AuNp	6.8	1.9	1.3	2.3	3.2	2.5	0.8	0.4	1.5	0.2

## Data Availability

Not applicable.

## Supplementary Materials

The following supporting information can be downloaded at: https://www.mdpi.com/article/10.3390/nano12183111/s1, Table S1: The latest method proposed for the assay of CA 15-3, CEA, and HER2; Table S2: Determination of CA15-3, CEA, and HER2 in whole blood samples.
