# Supplementary material for: Carbon Nanopowder-Based Stochastic Sensor for Ultrasensitive Assay of CA 15-3, CEA and HER2 in Whole Blood
Source: Nanomaterials (Basel). 2022 Sep 8;12(18):3111. doi: 10.3390/nano12183111 (PMC9501540; doi:10.3390/nano12183111)
Supplement: Supplementary file 1 [file nanomaterials-12-03111-s001.zip › nanomaterials-1856637-supplementary.pdf]

Supplementary materials

Article

Carbon Nanopowder-Based Stochastic Sensor for Ultrasensitive Assay of CA 15-3, CEA and HER2 in Whole Blood

Raluca-Ioana Stefan-van Staden [1,2,*], Oana-Raluca Musat [2], Damaris-Cristina Gheorghe [1], Ruxandra-Maria Ilie-Mihai [1] and Jacobus (Koos) Frederick van Staden [1,*]

1 Laboratory of Electrochemistry and PATLAB, 202 Splaiul Independentei Str., 060021 Bucharest, Romania
2 Faculty of Chemical Engineering and Biotechnologies, University Politehnica of Bucharest, 011061 Bucharest, Romania
* Correspondence: ralucavanstaden@gmail.com (R.-I.S.-v.S.); koosvanstaden2012@yahoo.com (J.F.v.S.)

Citation: Stefan-van Staden, R.-I.; Musat, O.-R.; Gheorghe, D.-C.; Ilie-Mihai, R.-M.; van Staden, J. (Koos) F. Carbon Nanopowder-Based Stochastic Sensor for Ultrasensitive Assay of CA 15-3, CEA and HER2 in Whole Blood. *Nanomaterials* **2022**, *12*, 3111. https://doi.org/10.3390/nano12183111

Academic Editors: Daniela Iannazzo and Simone Morais

Received: 24 July 2022
Accepted: 7 September 2022
Published: 8 September 2022

Publisher's Note: MDPI stays neutral with regard to jurisdictional claims in published maps and institutional affiliations.

Copyright: © 2022 by the authors. Licensee MDPI, Basel, Switzerland. This article is an open access article distributed under the terms and conditions of the Creative Commons Attribution (CC BY) license (https://creativecommons.org/licenses/by/4.0/).

Supplementary materials

Table S1. The latest method proposed for the assay of CA 15-3, CEA, and HER2.

**Biomarker**	**Tool**	**Linear concentration range**	**Limit of determination**	**Ref.**
CEA	Immunosensor	0.01pg mL^{-1} – 80.00ng mL^{-1}	10fg mL^{-1}	[6]
	Aptamer biosensor	1-30000 pg mL^{-1}	1pg mL^{-1}	[7]
	Graphene-based immunosensor	0.1 – 5.0ng mL^{-1}	0.1ng mL^{-1}	[8]
	Fe(TPFPP)Cl/AuNp	12.8pg mL^{-1}- 20.0µg mL^{-1}	12.8pg mL^{-1}	This work
	TPP/AuNp	100fg mL^{-1}- 1µg mL^{-1}	100fg mL^{-1}	This work
CA 15-3	Immunosensor	0.1fg mL^{-1} – 1.0µg mL^{-1}	0.1fg mL^{-1}	[9]
	Quantum dots/electrochemiluminescence	10µU mL^{-1}-500U mL^{-1}	10µU mL^{-1}	[10]
	Fluorescent biosensor	Up to 25.6µU mL^{-1}	-	[11]
	Fe(TPFPP)Cl/AuNp	100nU mL^{-1}- 1000U mL^{-1}	100nU mL^{-1}	This work
	TPP/AuNp	100nU mL^{-1}- 1000U mL^{-1}	100nU mL^{-1}	This work
HER2	Fiber-optic ball-tip resonator	3.7pg mL^{-1} – 128.0ng mL^{-1}	3.7pg mL^{-1}	[12]
	Bifunctional carbon nanorods	Up to 900pg mL^{-1}	7fg mL^{-1}	[13]
	Aptamer based colorimetric sensor	Up to 50.0pmol mL^{-1}	9.8pmol mL^{-1}	[14]
	Fe(TPFPP)Cl/AuNp	3.9fg mL^{-1}- 30.0pg mL^{-1}	3.9fg mL^{-1}	This work
	TPP/AuNp	35fg mL^{-1}- 39pg mL^{-1}	35fg mL^{-1}	This work

Supplementary materials

Table S2 Determination of CA15-3, CEA, and HER2 in whole blood samples.

Sample no	U mL^{-1}, CA 15-3			ng mL^{-1}, CEA			pg mL^{-1}, HER 2		
	Stochastic microsensors based on								
	Fe(TPFPP)Cl/ AuNp	TPP/AuNp	ELISA	Fe(TPFPP)Cl/ AuNp	TPP/AuNp	ELISA	Fe(TPFPP)Cl/ AuNp	TPP/AuNp	ELISA
1	448.09±0.03	447.52±0.02	450.00±0.12	24.00±0.05	23.18±0.02	24.00±0.12	64.43±0.02	64.40±0.05	63.00±0.17
2	448.12±0.03	449.49±0.05	443.00±0.17	7.18±0.05	7.21±0.03	7.30±0.10	4.96±0.02	4.90±0.03	4.87±0.12
3	206.55±0.05	205.30±0.03	204.00±0.14	17.87±0.04	17.98±0.03	17.00±0.12	61.97±0.04	62.03±0.05	60.00±0.10
4	265.72±0.03	269.13±0.04	260.00±0.12	17.15±0.04	17.78±0.02	16.98±0.13	64.39±0.03	64.22±0.02	64.00±0.11
5	398.64±0.03	398.78±0.02	392.00±0.14	11.80±0.03	11.85±0.03	11.23±0.14	40.94±0.02	39.14±0.05	38.40±0.12
6	448.10±0.03	428.15±0.08	440.10±0.12	13.90±0.04	13.96±0.02	13.15±0.11	33.01±0.02	32.25±0.03	33.10±0.14
7	220.87±0.03	223.15±0.04	221.00±0.15	3.20±0.02	3.19±0.03	3.00±0.14	18.04±0.02	18.00±0.03	17.45±0.13
8	563.86±0.07	569.43±0.03	560.0±0.11	11.14±0.02	11.85±0.03	12.00±0.11	3.36±0.04	3.76±0.05	3.12±0.02
9	303.88±0.04	304.20±0.03	300.00±0.15	12.00±0.03	11.79±0.02	11.50±0.14	27.97±0.03	28.03±0.05	25.50±0.12
10	111.35±0.03	118.40±0.05	115.20±0.14	8.50±0.02	8.43±0.03	8.50±0.12	28.51±0.03	28.28±0.02	27.50±0.11
t-test	2.13	2.21	-	2.61	2.29		2.41	2.38	-
